# Determinants for the humanitarian workforce in migrant health at the US-Mexico border: optimizing learning from health professionals in Matamoros and Reynosa, Mexico

**DOI:** 10.3389/fpubh.2024.1447054

**Published:** 2024-10-10

**Authors:** Christopher W. Reynolds, Savannah F. Ryan, Eesha Acharya, Ipek Berberoglu, Samuel Bishop, Brendon Tucker, Juan Daniel Barreto-Arboleda, Jorge Armando Flores Ibarra, Penelope Vera, Laura Jocelyne Fuentes Orozco, Sarah Draugelis, Amir M. Mohareb, Florian Schmitzberger

**Affiliations:** ^1^Department of Surgery, University of Michigan Medicine, Ann Arbor, MI, United States; ^2^University of Michigan Medical School, Ann Arbor, MI, United States; ^3^Department of Economics and Public Health, University of Michigan, Ann Arbor, MI, United States; ^4^Department of Surgery, Section of Plastic Surgery, University of Michigan Medicine, Ann Arbor, MI, United States; ^5^University of Pittsburgh School of Medicine, Global Response Medicine, Reynosa, Tamaulipas, Mexico; ^6^Universitetet i Oslo, Oslo, Norway; ^7^Pontificia Universidad Javeriana, Bogota, Colombia; ^8^Universidad Tamaulipeca, Reynosa, Tamaulipas, Mexico; ^9^Team fEMR, St Clair Shores, MI, United States; ^10^Center for Global Health, Massachusetts General Hospital, Boston, MA, United States; ^11^Department of Medicine, Harvard Medical School, Boston, MA, United States; ^12^Department of Emergency Medicine, University of Michigan Medicine, Ann Arbor, MI, United States

**Keywords:** humanitarian assistance, migrant health, refugee, US–Mexico border, global health, immigration, health worker shortages

## Abstract

**Introduction:**

Shortages of health professionals is a common problem in humanitarian settings, including among migrants and refugees at the US-Mexico border. We aimed to investigate determinants and recruitment recommendations for working with migrants to better understand how to improve health professional participation in humanitarian efforts.

**Methods:**

Semi-structured interviews were conducted with health professionals working with migrants at the US-Mexico border in Matamoros and Reynosa, Mexico. The study aimed to identify motivations, facilitators, barriers, and sacrifices to humanitarian work, and recommendations for effective learning approaches to increase participation. Participants included health professionals working within humanitarian organizations to deliver healthcare to migrants living in non-permanent encampments. Interviews lasted approximately 45 min and were analyzed in NVivo14 using a validated codebook and team-based methodology.

**Results:**

Among 27 participants, most were female (70%) with median age 32. Health professionals included nurses (41%), physicians (30%), logisticians (11%), social workers (7%), an EMT (4%), and a pharmacist (4%) from the US (59%), Mexico (22%), Cuba (11%), Peru (4%), and Nicaragua (4%) working for four organizations. Participants expressed internal motivations for working with migrants, including a desire to help vulnerable populations (78%), past experiences in humanitarianism (59%), and the need to address human suffering (56%). External facilitators included geographic proximity (33%), employer flexibility (30%), and logistical support (26%). Benefits included improved clinical skills (63%), sociocultural learning (63%), and impact for others (58%). Negative determinants included sacrifices such as career obligations (44%), family commitments (41%), and safety risks (41%), and barriers of limited education (44%) and volunteer opportunities (37%). Participants criticized aspects of humanitarian assistance for lower quality care, feeling useless, and minimizing local capacity. Recommendations to increase the health workforce caring for migrants included integration of humanitarian training for health students (67%), collaborations between health institutions and humanitarian organizations (52%), and improved logistical and mental health support (41%).

**Conclusion:**

Health professionals from diverse roles and countries identified common determinants to humanitarian work with migrants. Recommendations for recruitment reflected feasible and collaborative approaches for professionals, organizations, and trainees to pursue humanitarian health. These findings can be helpful in designing interventions to address workforce shortages in humanitarian migrant contexts.

## 1 Introduction

Health worker shortages are one of the most pressing problems affecting humanitarian and global health settings (1). This burden is exacerbated in low-middle income countries (LMICs), as 83 of 186 countries did not meet minimum health professional thresholds as outlined by the World Health Organization (2). While multiple components are needed for quality care delivery including adequate resources, access pathways, and political willingness, availability and capacity of health workers remains one of the hardest to solve in resource-constrained environments (3). With more than 100 million forcibly displaced persons worldwide depending on humanitarian health systems for necessary care (4), determining how to better motivate, recruit, and empower health professionals to work in such systems is imperative to optimizing migrant care (3). At the US-Mexico border, over 2.4 million migrants annually are seeking safe passage into the United States, with drastic increases in recent years (5). There, asylum seekers and refugees are living in non-permanent encampments lacking basic public health measures, face increased risks for disease and violence, and depend on humanitarian organizations for basic medical care (6). Despite increased health needs and potential acuity of vulnerable migrants at the US-Mexico border, there is a dearth of health professionals working with this population (6).

It is unclear why health worker shortages remain such a persistent problem in humanitarian and other low-resource health contexts (7, 8). Some factors are well-known, including a lack of compensation, fear of burnout or emotional exhaustion, and concerns for safety (8–11). In 2023, 2,562 incidents of violence against health professionals working in conflict settings were reported across 30 countries, a 25% increase from the previous year (12). Additional hypotheses include a lack of humanitarian career development pathways and limited opportunities for education among health professional students (13). Studies aimed at investigating motivations for humanitarian health work have demonstrated that religious and moral motivations, self-perceived usefulness, increased compensation, training opportunities, and leadership development can all incentivize health workers to work in these contexts (8, 14, 15). Some efforts have explored the impact of medical education initiatives to address health worker shortages in underserved areas, including exposing medical students to global health, service-learning opportunities, and increasing admissions of students from these areas (16, 17). However, many of these efforts focus specifically on rural or global health and fail to account for the specific characteristics of humanitarian contexts including safety risks, acute mobilization, and shorter-term commitments (17). Even less is understood about health worker motivations to care specifically for migrants in humanitarian settings, including refugees and asylum seekers at the US-Mexico border (18).

In this study, we aimed to investigate the determinants which motivate health professionals to work with migrant populations at the US-Mexico border, as well as recommendations for motivating others to address health workforce shortages among refugee populations. Specifically, we sought to understand the internal motivations and sacrifices, external facilitators and barriers, and challenges of humanitarian aid experienced by health professionals caring for migrants at the US-Mexico border. These findings could prove valuable in understanding best practices to promote facilitators, mitigate barriers, and enhance recruitment of qualified health professionals to improve health worker availability for vulnerable migrant populations.

## 2 Methods

### 2.1 Study design

To determine the motivations, facilitators, sacrifices, barriers, and recruitment strategies for humanitarian health professionals, a qualitative study with a phenomenological approach was undertaken. This methodology was considered most appropriate given the complex internal and external, individual-centered factors involved in the study question and the opportunity to elicit detailed explanations provided by qualitative methods (19). First, a semi-structured interview script was created following a literature search of qualitative studies examining the experiences of health professionals working in migrant and other humanitarian contexts (20). Preliminary questions were content validated by administering the interview and receiving feedback from health professionals working at the US-Mexico border (*n* = 4). Following clarification of questions and purging repetitive items, the final interview script was designed to last 45 min and contained 25 items covering participant characteristics, incentives and disincentives, and tangible solutions to address health workforce shortages (Supplementary material 1).

### 2.2 Study setting and participants

This study took place at the US-Mexico border in Matamoros and Reynosa, Mexico. These study sites were selected as they are two of the major points of entry for those seeking asylum in the United States, and where migrant encampments have been established following changes to US immigration policy beginning in 2019. In these locations, migrants including asylum seekers and refugees live in non-permanent encampments with limited access to public health measures and face increased exposure to disease and violence (6). Humanitarian non-governmental organizations provide most health and other social services to migrants in these locations, while asylum seekers await processing into the US for indeterminate amounts of time (21). Global Response Medicine (GRM) and Médicos Sin Fronteras (MSF) are two of the major organizations providing physical and mental health services, respectively, to these populations in Matamoros and Reynosa (22). Eligible participants for this study included health professionals, defined as those delivering clinical care or health services to migrants residing within these camps. Such professionals included physicians, nurses, emergency medical technicians, logisticians, social workers, physical therapists, and clinical psychologists. Participants spoke English, Spanish, or both and were required to have worked with migrants for at least 1 week. Lawyers, paralegals, legal advocates for asylum seekers, and others without formal health professional training or limited clinical experience with migrants were excluded from this study.

### 2.3 Data collection

The first round of interviews was conducted in Matamoros, Mexico in the main migrant encampment in July 2020. The second round was conducted in February 2023, in Reynosa, Mexico in four camps located throughout the city. At both locations, a snowball sampling method was utilized to recruit participants with diverse ages, roles, and humanitarian experiences. First, investigators approached health professionals from GRM and MSF within the migrant camps and at established clinical sites. These participants were asked to recommend additional eligible persons from other humanitarian organizations who may be interested in participating. All interviews were administered by a bilingual, qualitative researcher who was not affiliated with a local humanitarian organization, and in a private location in English or Spanish according to patient preference. Interviews were audio recorded with the EasyVoiceRecorder app on iPhone and uploaded to a secure DropBox (Dropbox, Inc.; San Francisco United States) only available to study researchers. Interviews were continued until data saturation was reached, defined as the cessation of new themes according to preliminary analysis of the study team.

### 2.4 Data analysis

**Figure 1:**
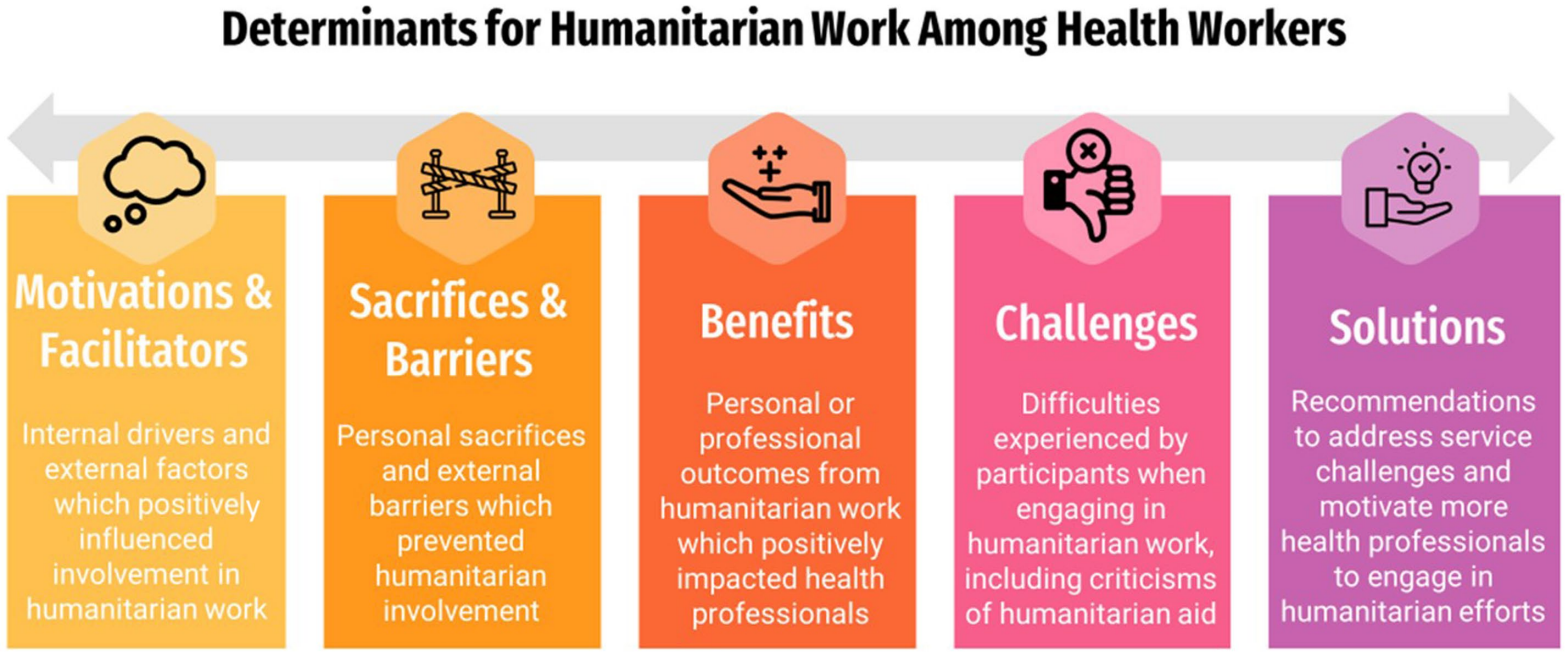
Major themes describing determinants to work with migrants in humanitarian contexts among health professionals in Matamoros and Reynosa, Mexico.

Participants were assigned a numeric code to further guarantee anonymity and interviews were transcribed verbatim in Microsoft Word. Analysis was conducted using a team-based content analysis approach with a validated codebook (23). Specifically, two researchers underwent inductive immersion into the transcripts to develop a codebook with major themes, which then underwent an interactive process of independent coding, calculating interrater agreement, and refinement to clarify themes (Supplementary material 2). Following three rounds of iteration, a Cohen’s Kappa of 0.79 (*κ* = 0.79) was achieved and considered adequate (24). The final codebook contained 61 subthemes categorized into seven major themes: motivations, facilitators, benefits, sacrifices, barriers, challenges, and solutions (Figure 1). Transcripts were coded in NVivo14 (QSR International, Burlington, MA, United States) and theme frequencies were calculated. Quotes were selected to represent a diverse range of participants and translated into English. Measures were taken to ensure trustworthiness of data including (i) using a team-based and validated coding method, (ii) independent and cooperative data analytic techniques by multiple study members, (iii) member checking with health professionals on the research team, and (iv) consensus on final results by research members.

### 2.5 Ethical compliance

This study adheres to SRQR guidelines for qualitative research (25).

## 3 Results

### 3.1 Participant characteristics

**Figure 2:**
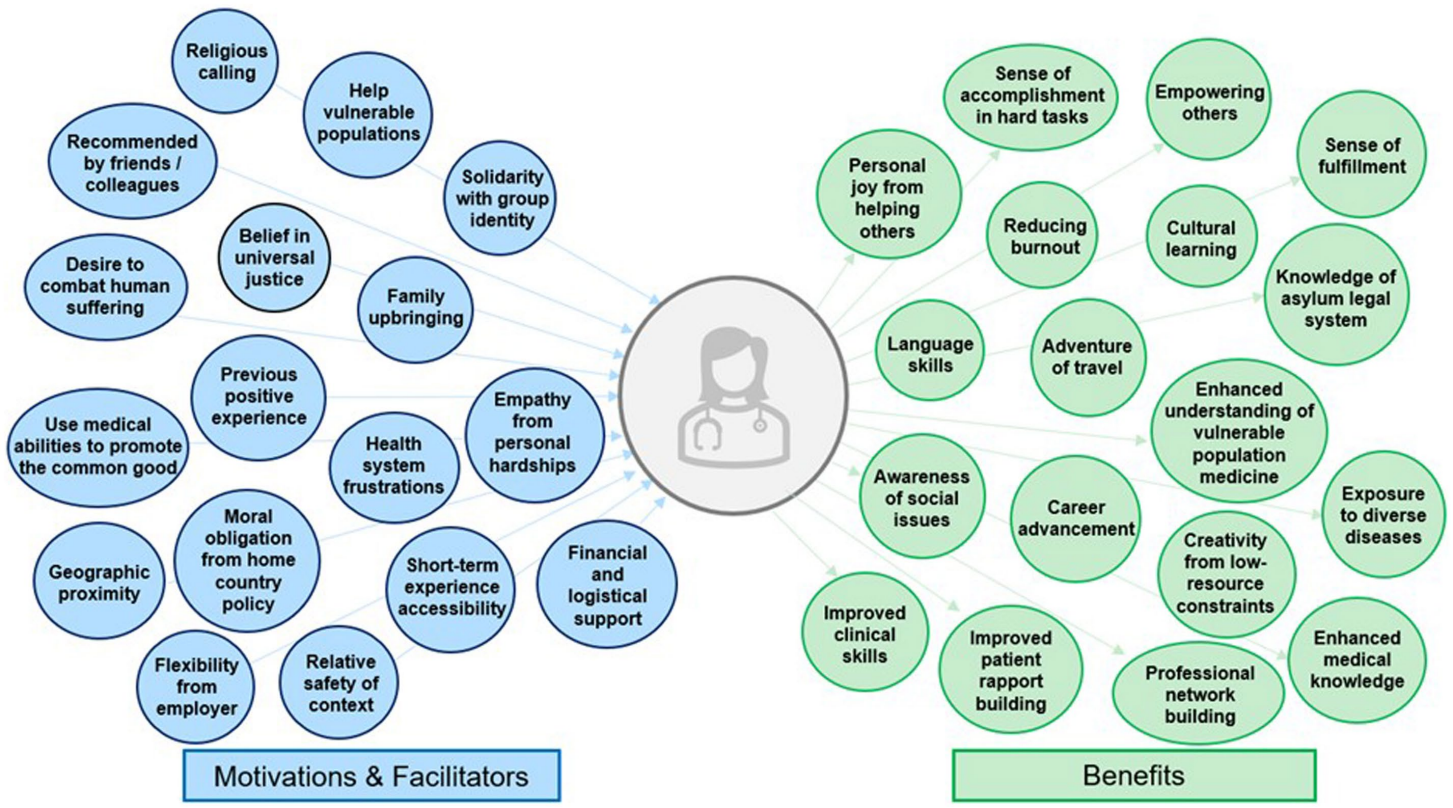
Subthemes of internal motivations, external facilitators, and benefits to working with migrants in humanitarian contexts among health professionals in Matamoros and Reynosa, Mexico.

Twenty-seven interviews were conducted among humanitarian health workers in Reynosa (*n* = 17) and Matamoros, Mexico (*n* = 10; Table 1). Participants were mostly female (70%), with ages ranging from 22 to 67 years old (median = 32, IQR = 29–54). Participants hailed from the United States (59%), Mexico (22%), Cuba (11%), Peru (4%), and Nicaragua (4%), with varying levels of education and professional roles. Two-thirds performed patient consultations: “*I am a general physician. So what I normally do is consult any patient that arrives: chronic patients, children, well-child visits, prenatal care, or whatever arrives, I administer treatments or any other procedure you would do in a consult”* -Physician. Other roles included doing triage and patient intake (11%), providing psychosocial services (15%), logistics management (19%), interpreter services (4%), and pharmacy management (4%). There was a mix of short-term volunteers (41% <1 month) and long-term humanitarian workers (26% >1 year), with many having experience in other humanitarian settings (74%) and specifically with asylum seeker populations (41%). Participants from Matamoros had a higher likelihood of working in the camp for a longer period of time (>6 months) than those from Reynosa (*p* = 0.049; Figure 2).

**Table 1:** Participant characteristics among humanitarian health workers in Reynosa and Matamoros, Mexico.

	Total *n* = 27 (%)	Reynosa *n* = 17 (%)	Matamoros *n* = 10 (%)
**Gender**			
Male	8 (30%)	3 (18%)	5 (50%)
Female	19 (70%)	14 (82%)	5 (50%)
**Age**			
18–25	4 (15%)	4 (24%)	0 (0%)
26–40	12 (44%)	7 (41%)	5 (50%)
41–65	8 (30%)	5 (29%)	3 (30%)
> 65	3 (11%)	1 (6%)	2 (20%)
**Country of origin**			
USA	16 (59%)	11 (65%)	5 (50%)
Mexico	6 (22%)	6 (35%)	0 (0%)
Cuba	3 (11%)	0 (0%)	3 (30%)
Peru	1 (4%)	0 (0%)	1 (10%)
Nicaragua	1 (4%)	0 (0%)	1 (10%)
**Highest education**			
Terminal degree (PhD, MD, DNP)	8 (30%)	5 (29%)	3 (30%)
Master’s (NP, MS, MPH)	11 (41%)	7 (41%)	4 (40%)
Bachelor’s	7 (26%)	4 (24%)	3 (30%)
Associates	1 (4%)	1 (6%)	0 (0%)
**Occupation**			
Nurse	11 (41%)	8 (47%)	3 (30%)
Physician	8 (30%)	5 (29%)	3 (30%)
Logistician	3 (11%)	1 (6%)	2 (20%)
Social Worker	2 (7%)	2 (12%)	0 (0%)
Interpreter	1 (4%)	0 (0%)	1 (10%)
Pharmacist	1 (4%)	0 (0%)	1 (10%)
Emergency Medical Technician	1 (4%)	1 (6%)	0 (0%)
**Work with humanitarian organization**			
Patient consultations	18 (67%)	12 (71%)	6 (60%)
Triage and patient intake	3 (11%)	2 (12%)	1 (10%)
Psychosocial services	4 (15%)	2 (12%)	2 (20%)
Logistics management	5 (19%)	3 (18%)	2 (20%)
Interpreter services	1 (4%)	0 (0%)	1 (10%)
Pharmacy management	1 (4%)	0 (0%)	1 (10%)
**Time working with current humanitarian organization**			
< 1 month	11 (41%)	7 (41%)	4 (40%)
1–6 months	5 (19%)	5 (29%)	0 (0%)
7–12 months	4 (15%)	0 (0%)	4 (40%)
> 1 year	7 (26%)	5 (29%)	2 (20%)
Worked in asylum health in the past	11 (41%)	7 (41%)	4 (40%)
Worked in humanitarian health in the past	20 (74%)	12 (71%)	8 (80%)
**Time working in humanitarian medicine over lifetime**			
< 1 month	9 (33%)	7 (41%)	2 (2%)
1–6 months	3 (11%)	3 (18%)	0 (0%)
7–12 months	1 (4%)	0 (0%)	1 (10%)
> 1 year	14 (52%)	7 (41%)	7 (70%)

### 3.2 Motivations

Participants identified internal motivations to pursue humanitarian health work with migrants. Most commonly, participants expressed a personal desire to help vulnerable populations (78%; Table 2). There were multiple components of personal identity which motivated participation, including a belief in universal justice (41%), using one’s skills and privilege as a health professional for good (33%), a religious calling (11%), and family upbringing which encouraged service work (19%). Others expressed having a high level of personal resilience (7%), which they believed obligated them to humanitarian work given their unique persistence. Empathy was commonly mentioned, as participants witnessed the intense suffering of refugees (56%) or had undergone their own hardships (15%). Two providers from Matamoros who were asylum seekers themselves felt an especially deep form of solidarity and call to serve this population (7%). Some had positive experiences in the past with humanitarian or disaster relief work which inspired them to seek further opportunities (59%), while others were motivated by recommendations from colleagues and mentors (33%). Participants reported being frustrated with their home health systems (19%), which prioritized efficiency, revenue generation, and protocolization above care for those needing it most. These professionals hoped that doing humanitarian work could provide an antidote to burnout and recenter their original motivations for pursuing a health career: to care for others. Some motivations were unique to work at the US-Mexico border. US participants expressed a moral obligation to care for migrants at their Border, as they attributed the vulnerable health status and residence in camps of migrant patients to restrictive US immigration policy, which they felt responsible to reconcile (11%).

**Table 2:** Internal motivations and external facilitators to work with migrants in humanitarian contexts among health professionals in Reynosa and Matamoros, Mexico.

Theme	Count (%)	Representative quote
**Internal motivations**		
Desire to help vulnerable populations	21 (78%)	“I like the idea of helping people that have nothing. I feel comfortable and enjoy it. It does not feel like work, but something natural.” -Logistician
Past positive experience in humanitarian work	16 (59%)	“I think my previous experience definitely makes me more likely work with asylum seekers. I felt a huge push from past experiences for the passion to be in places like this and do this work.” -Nurse
Witnessing human distress and suffering	15 (56%)	“There are people who do not deserve the treatment they receive. I’ve seen cases of rape, abuse, and mistreatment that breaks your heart listening to these stories.” -Physician
Belief in justice and fairness	11 (41%)	“I think that everyone with privilege should realize the needs of others and work with vulnerable persons. Once you meet these inspiring people, you realize this is where I should be working.” -Social worker
Obligation to use professional skills for common good	9 (33%)	“It’s a justice issue. If you have the ability as someone in healthcare to alleviate another’s suffering, then you have an obligation to do so.” -Nurse
Recommendations from friends or colleagues	9 (33%)	“One night my attending [physician] happened to be the director of GRM, and she mentioned something about humanitarian work so I inquired and learned about it from there.” -Nurse
Family upbringing	5 (19%)	“I grew up with parents talking about how lucky we are. There are people who aren’t as fortunate and I want you to see and interact with them. If you are that lucky, you should help them.” -EMT
Religious calling	3 (11%)	“I’ve lived here my whole life and this is where God has me now. My skills and talents are being used where God thinks is best.” -Logistician
Frustration with home health system	5 (19%)	“The health system in the US is so focused on making money. I just want to help people and all this humanitarian work, especially with migrants, reminds you to help those without consistent care.” -Nurse
Empathy from personal hardship	4 (15%)	“It is easy to feel connected because I hear their traumas and feel so bad because I was feeling bad for my problems, but they do not compare.” -Interpreter
Moral obligation related to home country’s restrictive policies	3 (11%)	“I’ve been upset and sad about the behavior of our country towards our neighbors. And so as a physician, one of the ways I try to make restitution for our sins is to help out medically.” -Physician
High level of resilience	2 (7%)	“Living through my own challenges, I know what my capacities are. I can tolerate beyond what the average person can. So if I can do that and have the clinical skills, I should help.” -Nurse
Solidarity with group identity	2 (7%)	“I am one of them. I am a doctor, but also am a migrant asylum seeker. I’ll never lose that part of myself, and feel a great solidarity to relate myself more to these patients.” -Physician
**External facilitators**		
Geographic proximity to volunteer site	9 (33%)	“I started working because it was accessible. I was already staying in the area, and the project manager knew of my skills and asked if I wanted to help.” -Interpreter
Permission from employer	8 (30%)	“The fact that my hospital offers support to be here is pretty outstanding. They have a flexible enough schedule that I can work a little extra then take time off as a block and have coverage for those two weeks.” -Physician
Logistical support	7 (26%)	“It normal takes a lot of money to travel. But GRM now has Airlink to cover travel expenses and a guest house, which is unheard of for work like this, but makes it much more financially accessible.” -Nurse
Flexibility in work life, career change, or unemployment	6 (22%)	“Originally I was travel nursing and finished an assignment in April. I decided it was a good time to come because I had some savings and no work commitments.” -Nurse
Relative safety of context	6 (22%)	“There’s all these issues around the world of much bigger scale: Syria, Yemen, Sudan, and they are inaccessible for our hospital from a safety and security perspective. The border has safety challenges but is a more chronic problem with known concerns.” -Nurse
Financial support	5 (19%)	“I do not have a barrier because my hospital is taking care of everything financially. Even my airfare was covered, either by the organization or my employer.” -Nurse
Short-term experience	5 (19%)	“I had always been interested in this work, but many of the well-known groups like Doctors without Borders and UNICEF require extreme commitments like a minimum six months commitment. This commitment is much easier, because I can live in the states and volunteer for one week or two.” -Nurse
Working within comfort of personal capacity	3 (11%)	“One of my personal concerns is personal safety. I’m not going to go to a war zone unless I’m trained to do that, so I was glad to find something where I could contribute without putting myself in harm’s way.” -Physician

### 3.3 Facilitators

Participants expressed external facilitators which allowed them to do humanitarian work. The greatest facilitator was employer related. Among full-time humanitarian workers, they appreciated having employment which allowed them to use their skills while addressing a social problem. For short-term volunteers traveling from the US, employer support was a key facilitator (30%). In some cases, clinicians received salary or logistical support for their humanitarian work (19%), facilitated by employer-sponsored disaster, humanitarian, or global health institutes with the funding and flexibility to allocate this time. Participants in Reynosa were more likely to mention employer support, as many worked for institutions with established partnership to GRM’s Reynosa project. More commonly, participants sacrificed personal vacation time, were between jobs, or intentionally structured their careers with the freedom to volunteer, such as the case among travel nurses or career humanitarians (22%). A key facilitator even for seasoned humanitarians was logistical support from the organization, as GRM provided free housing and travel (26%). For those sacrificing vacation or salary, this support proved financially and emotionally beneficial. Finally, the US-Mexico border context yielded additional facilitators leading to commitment to do this work. Participants expressed that most organizations require commitments of months to years to do humanitarian work, place workers in distant and unfamiliar contexts, and may require willingness to work in conflict and other unsafe areas. While participants recognized safety concerns at the US-Mexico border, particularly with the presence of cartels, they felt that the opportunity to volunteer for shorter amounts of time (19%), proximity to home for US clinicians (33%), and relative safety (22%) increased their ability to dedicate time to this work.

### 3.4 Benefits

Health professionals identified multiple benefits to working with migrants. First, most expressed feeling a personal joy, fulfillment and increased sense of purpose from helping others and making a positive difference (58%): “*It feels good to know you have helped someone, knowing you were a face that made them seen and valued despite what the rest of the world is telling. I enjoy being able to affirm people’s value in that way*.” -Nurse. Others felt accomplished recruiting and empowering others to care for vulnerable migrants (22%): “*The most satisfying piece is enabling people to do this work for the first time and in a professional, respectful, appropriate way. My hope is they go off and find a piece of this world which inspires them to keep doing this work*.” -Nurse. Many appreciated the adventure of travel (22%) and increasing their network of colleagues with similar values and career interests (44%). Most benefited from learning about new cultures, as asylum seekers with diverse beliefs arrived from all over the world, particularly Latin America and the Caribbean (63%): “*The biggest benefit is meeting people from all over the world. Listening to their stories and having friendships from other countries including with volunteers. It is beautiful to experience new places through the stories of others”* -Logistician. Four clinicians even improved their Spanish language skills through patient consultations and collaboration with Mexican physicians (15%).

US workers gained a heightened awareness of social issues, particularly the politico-legal dimensions of immigration and asylum. 37% felt that their work with asylum seekers changed their perception on the best way to deliver care to vulnerable groups, including the importance of psychosocial issues, preventive care, and empowering migrant populations by involving them in health service co-design. Participants reported new clinical skills from the difficulty of this work, including caring for patients requiring extensive social services, treating diseases which were uncommon at home, and using creativity to provide quality care in a resource-constrained environment (63%). They reported improved care delivery methods and ways of relating to patients, particularly when language and resource barriers impaired care. Clinicians believed working with migrants made them better health professionals for their improved clinical skills, medical knowledge, and rapport-building: *“I’ve gained so much knowledge, especially working in this context. When I started, I had a ‘school ideology’ based in North American textbooks but have learned how to integrate needed skills and learning. I’m always seeing the sickest patients, so that pushes me to do all I can for them. It has helped me grow into a better clinician”* -Physician.

### 3.5 Sacrifices

Participants also counted personal sacrifices which disincentivized them from humanitarian work. Most frequently mentioned were career obligations, as many found it difficult to sacrifice time from work or devote vacation to humanitarian work (44%). While many reported improved patient skills, most employers did not value humanitarian work in a way that facilitated career rewards. Physicians more frequently mentioned this struggle when compared with other health professionals, as time away had to be compensated by practice partners. Participants reported financial sacrifices, including forgoing salary and self-funding travel and lodging to do this work (26%). Family commitments were second most common (41%). First-time volunteers expressed wanting to work with migrants earlier but not wanting to leave children or partners to do so. Long-term humanitarian professionals found it harder to establish long-term connections to a home base area and described the challenge of living a nomadic lifestyle (11%). Almost half of participants sacrificed safety and comfort for humanitarian work, causing nervousness when becoming involved (41%). Finally, one-third listed emotional difficulties, including witnessing intense human suffering and stark health inequities, leading to an emotional burden and occasional burnout (33%). One-third of participants explicitly stated they encountered no or negligible sacrifices to their humanitarian work (33%). Those living close to the border including Mexican physicians, asylum seeker health workers, and US clinicians residing in border states were more likely to report having no sacrifices to humanitarian work.

### 3.6 Barriers and challenges of humanitarian aid

Alongside personal sacrifices, participants cited external barriers to humanitarian health work, including overall criticisms of the field. Barriers included limited knowledge, education, and career development in humanitarian activities (44%). From the time of being students to fully certified clinicians, participants struggled to learn about, apply, and be accepted to volunteer with assistance groups (37%). Most actively sought out extracurricular or service-oriented volunteering but discovered limited long-term career development opportunities, as few planned academic or clinical careers around humanitarian work. Certain aspects of clinical work challenged participants’ effectiveness, including language barriers with primarily Spanish and Haitian Creole speaking patients (11%), limited autonomy depending on local delivery structures (7%), and unfamiliar protocols with different standards of care from typical practice (19%). Health professionals criticized aspects of humanitarian aid generally, including that organizations did not track reliable patient care metrics (7%), had limited resources and funding (19%), and created tensions between migrant community expectations with feasible resource provision (7%). Some professionals believed that quality standards were being lowered due to a lack of resources and objective accountability (22%). Five worried that the presence of humanitarian organizations could unintentionally minimize the capacity of local communities and governments to help themselves, particularly when done without equitable partnership (19%). Many worried that the rapid turnover and varied clinical specialties of volunteers limited consistency and standardization. Finally, participants expressed frustration with bureaucracy of humanitarianism (15%), the prioritization of appearances over quality work (15%), and conflicts between overlapping organizations (7%). Overall, first-time volunteers were just as likely to criticize humanitarian aid as experienced participants, as there was no association between the number of previous humanitarian experiences and likelihood of a participant to share critiques (Table 3).

**Table 3:** Perspectives on internal sacrifices, external barriers, and common challenges to humanitarian assistance from health professionals in Reynosa and Matamoros, Mexico.

Theme	Count (%)	Representative quote
**Internal sacrifices**		
Career obligations	12 (44%)	“The problem is you need to be available to deploy on short notice, and most employers aren’t down for that. You cannot tell your boss you are taking two weeks off tomorrow.” -Nurse
Family commitments	11 (41%)	“With responsibilities from a full-time job, family, pet, or parenting, it is hard to break away from all that to do something.” -Nurse
Risks to safety and comfort	11 (41%)	“All the horrible things, even being in Reynosa, you have to have a heightened safety awareness because of cartels and the real danger they pose. It can expose you to horrible things in the world.” -Nurse
Emotional difficulties including witnessing suffering	9 (33%)	“The greatest challenge is it hurts my heart. It’s hard to see people, especially children, in these conditions without food or security. They have suffered so much, and I try to love them as much as I can.” -Nurse
No perceived sacrifices	9 (33%)	“This has not been a sacrifice. I live in Reynosa, so I’ve enjoyed giving back to the community.” -Physician
Financial cost and lower salary	7 (26%)	“I think humanitarian work tends to be mobile and does not pay well. Those are some barriers, lifestyle of limited money and being away from home.” -EMT
Limited stability in personal life	3 (11%)	“I’m kind of living a nomadic life, with storage in one place, apartment in another, and car somewhere else. I miss being able to be in one area and have a home, but it comes with the territory of this much travel.” -Physician
**External barriers**		
Limited education and training	12 (44%)	“Most people just do not know. In school, I had a professor that spent two weeks each year medical volunteering, but that was the only thing I ever heard.” -Nurse
Limited volunteer opportunities	10 (37%)	“The organizational piece to get here was so hard. It took me 3 years of planning just to volunteer for a week. If I have a skill set, why is it so hard to find opportunities? You cannot figure out the right people to talk to or groups to work with” -Nurse
Language barriers	3 (11%)	“The language barrier was a big challenge. Interacting with English-speaking volunteers and with a completely different culture, being at the Border for the first time can be strange.” -Logistician
Already overburdened clinicians	2 (7%)	“Helping is very limited due to time. Most Mexican doctors have three different jobs, so they become robots in the hospital who are there 24/7. It does not give much time for altruistic work.” -Physician
Limited autonomy	2 (7%)	“Having to choose how to utilize my time. If I’m here, I’m not there, which limits me to focus on priorities.” -Logistician
**Challenges of humanitarian aid**		
Provider feelings of uselessness	6 (22%)	“The emotional burden comes from seeing so much and feeling hopeless, when there’s so many things you want to do but cannot. Seeing some sick kid without shoes when it’s 40 degrees out and we cannot even find him socks. You feel helpless when you cannot provide the care they need.” -Nurse
Lower quality care	5 (19%)	“You cannot really offer consistent care without resources to be effective in these situations. The resources are so limited we are treating symptoms when they really need something like respiratory treatment or an entire workup with labs. At the end of the day, we just do not have the access needed for them.” -Nurse
Funding limitations	5 (19%)	“Most of us are volunteers. I’ve made it clear my contribution is voluntary, but most people cannot do this work without economic remuneration.” -Logistician
Minimization of local capacity	5 (19%)	“You look at places which suffer the worst disasters like Haiti which has tons of disaster dollars poured in. And they are still doing terribly, because we go in and strip the capacity of local people to respond to their own emergencies.” -Nurse
Protocol differences	5 (19%)	“Learning what the typical treatment plan algorithm where you are working is difficult. There’s things the local staff does which are different than at home.” -Nurse
Prioritization of appearances	4 (15%)	“The hospitals like to have a disaster group because it looks good for them, but I do not know that they want a lot of people doing it because it probably does not make money. So that’s a tension.” -Physician
Bureaucracy	4 (15%)	“I wanted to work with migrant populations, though the bureaucracy of it all is complicated. I’ve learned that with bureaucracy, you cannot do much. So I sought out an organization which limited that so I could do something.” -Social worker
Burnout	3 (11%)	“When people are finally in positions of power, I feel they get burned out. So new volunteers come in bright eyed and bushy tailed but are trying to work under people who have been there so long they are jaded or have low expectations of what they can actually provide.” -Nurse
Secondary trauma	3 (11%)	“There is secondary trauma that happens with doing this work that can lead you to be harmed, either emotionally, psychologically, or physically. You put yourself in a dangerous way, it can lead to depression or moral injury.” -Physician
Poor data collection metrics	2 (7%)	“There’s a huge lack of quantifiable data on patients. No one has been doing research, which means they will not get the funding they deserve. They need to quantify the health issues related to limited clean water, or engage with patients to figure out the health barriers.” -Nurse
Balancing community expectations with feasibility	2 (7%)	“They wanted certain foods like chicken, but there’s no refrigeration. And we do not want people getting sick from rotten food. It’s a constant balance of how to allocate resources.” -Logistician
Interorganizational conflict	2 (7%)	“We developed a great plan, except it wasn’t accepted. It was just bypassed because honestly I think the Mexican officials from Instituto Nacional de Migracion felt like we were overstepping our boundaries.” -Logistician

### 3.7 Recommended solutions

Participants recommended solutions to better motivate, support, and recruit health professionals to humanitarian health delivery for migrants. Solutions generally mirrored the sacrifices, barriers, and challenges identified by participants (Table 4; Supplementary material 3). First, participants highlighted the need for active recruitment and collaboration of health professionals and students (67%). Some believed that humanitarian electives should be a requirement to obtain a professional health degree. Many believed that pathways for career humanitarians should be better defined and that humanitarian clinical or research opportunities should be made accessible for health professionals working at private and academic medical centers (22%). Second, participants recommended fostering partnerships between aid organizations, US medical institutions, professional societies, and health education schools (52%). Such connections could provide mutual benefit by facilitating volunteering, demonstrating to younger trainees examples of long-term careers in humanitarianism, fostering research and fundraising collaborations, mitigating employer-related barriers for practicing clinicians, and providing mentors to trainees and young health professionals interested in this work. Participants emphasized the importance of actively encouraging colleagues to become involved, as this was an influential factor for many of them (59%). They recommended doing so with an interdisciplinary approach, where social workers, public health professionals, advocates, interpreters, and others outside of clinical care could contribute valuable skills to migrant care. Third, participants emphasized the importance of demonstrating the health needs of their migrant patients (67%). They believed that exposing this reality to their colleagues and trainees would motivate those who were previously unaware to become involved. Fourth, participants encouraged aid organizations to take steps to mitigate the uncertainties and nervousness of first-time volunteers by providing comprehensive information including travel logistics, role descriptions, and safety protocols prior to applying for a volunteer role (26%). Fifth, health professionals recommended improving knowledge and accessibility of humanitarian work by highlighting opportunities beyond premier, well-known organizations such as the United Nations and Médecins Sans Frontières (Doctors Without Borders), which can be restrictively competitive due to popularity (30%). Specifically, they suggested that smaller organizations engage in writing and publishing activities or provide examples of creative approaches from volunteers who successfully navigated commitments of work or home to participate (22%). Finally, they encouraged organizations to support humanitarian workers through mental health support services, logistical support including for housing and travel, and institutionalized mechanisms to identify and mitigate burnout (41%).

**Table 4:** Recommended solutions to handle challenges of humanitarian assistance among migrants in Reynosa and Matamoros, Mexico.

Sacrifices, barriers, or challenges	Potential solutions	Representative quote
Burnout and secondary trauma	Provide mental health or spiritual support services such as chaplains	“There definitely needs to be a piece of this work where you are in a community where people acknowledge the secondary trauma that can happen. I actually trained to be a chaplain for the disaster team. And one of the experiences we had on assignment was so awful, I felt that support was needed and appreciated.” -Physician
	Allow decompression opportunities following traumatic experiences	
	Foster strong communities where secondary trauma can be processed	
Career obligations	Institutionalize humanitarian work opportunities through employers	“I do not create the motivation, rather facilitate it from a professional perspective. If I can wedge out a bit of our employees’ time and professional responsibilities and keep them getting paid, I can create a safety net to bring in all kinds of people who normally would not work in these settings.” -Nurse
	Promote leadership which will dedicate time to humanitarian work	
	Select a job in humanitarianism	
	Select a practice with multiple and flexible partners who can cover	
	Negotiate time for humanitarian work into one’s contract	
Family and social obligations	Pursue humanitarian work when commitments are minimal	“I’ve always wanted to explore a humanitarian project, but timing wise, I could not do this until my kids were out of the house. And then this opportunity appeared, where there was a need in my own backyard, so I jumped at it.” -Physician
	Select opportunities which mitigate tension between family and work	
	Involve families in work, where appropriate	
Financial burden	Provide salary coverage	“The big barrier for me was financial assistance to come here. So that’s a huge incentive if things like airfare or housing can be provided. It could be done in conjunction with some sort of commitment, like if we pay for X amount of your trip, then you’ll give us three week of volunteer time per year.” -Nurse
	Offer logistical support in covering travel or lodging expenses	
	Formalize volunteer time commitments for logistical support	
Ignorance to humanitarian needs	Write or publish stories, blogs, or research about humanitarian need	“I think it would be great to do medical campaigns, show people what is really happening here. Because most doctors do not actually know what is occurring. They just say ‘oh, they are migrants, but they already have help,’ when really that’s not true.” -Physician
	Demonstrate need to friends and colleagues with stories and photos	
	Offer short-term medical outreach campaigns	
Limited knowledge, education, and career development pathways	Collaboration between health schools and humanitarian organizations for clinical rotation or research opportunities	“It needs to be readily available in education. Having a professor that has done humanitarian work, who students can speak to and hear from someone whose actually done it and has knowledge on the subject. And then if students want to, opportunities could be created. If an organization partnered with a school, they could schedule a clinical rotation to give that student a very real, hands-on experience to get their feet wet. If there was more of an emphasis on it in schooling, it would not be such an unknown path forward or difficult thing to do.” -EMT
	Hiring health education faulty with humanitarian expertise	
	Integrate humanitarianism into medical education	
	Design curricula and service-learning opportunities for health students	
Limited opportunities	Actively recruit colleagues with well-suited skills	“One way is to increase recruitment. Something like providing lectures, or contacting organizations like the American college or any national society and letting people know these opportunities are available. Because I know many health professionals who want to do this work but they do not know about how to go about it or the right organizations to contact. Even newspapers, or paying to get a marketer. And for us, doing all we can to get the word out to others we work with. -Nurse
	Promote volunteering with smaller organizations	
	Recruit through national societies	
	Present humanitarian experiences to social networks	
	Create organizational partnerships	
Provider feelings of uselessness	Take time to develop clinical, humanitarian, and language skills before volunteering	“When I started my new job, I felt that I finally developed a baseline skill set in clinical care. But that’s one thing, and the entire other skill set in the humanitarian sphere is a whole other. I started to volunteer with our disaster response team.” -Nurse
	Align volunteer skills with community needs	
	Provide resources for adequate care delivery	
Risks to safety and comfort	Transparency on role and day-to-day activities	“One improvement would be more transparency, what a typical day looks like with responsibilities. I think the unknown scares people away. If you say I’m going out of the country for a humanitarian medical trip, people think it’s crazy. They think its so unsafe. If we can make the work more transparent, it would definitely get more interest.” -Nurse
	Well-designed safety protocols	
	Working in less risky environments	

Barriers in Table 4 were intentionally selected to reflect personal barriers for health professionals and those relating to the most commonly mentioned recruitment strategies. A full list of sacrifices, barriers, and challenges with corresponding solutions and representative quotes is available in Supplementary material 3.

## 4 Discussion

In this qualitative study of health professionals at the US-Mexico border, our sample identified common positive and negative determinants for working with migrant populations, as well as recommendations to address health worker shortages in migrant humanitarian contexts. Positive factors included internal motivations, external facilitators, and personal and professional benefits including a desire to help vulnerable populations, career flexibility, improved clinical skills, and a sense of joy and fulfillment from their work. Negative measures reflected internal sacrifices and external barriers such as career and family commitments, safety risks, limited education and volunteer opportunities, and criticisms of humanitarianism in general. Considering these determinants, participants suggested several strategies to increase recruitment of health professionals caring for migrants in humanitarian settings. These recommendations, which aimed to enhance benefits and mitigate challenges, could provide feasible and attainable solutions for organizations, individuals, and health employers to address health workforce shortages in humanitarian settings.

Positive determinants reflected concepts of altruism including a desire to help and vocation for justice, positive past experiences of previous volunteering or colleagues’ recommendations, and personal identity through group affiliation, family values, or moral responsibility based on citizenship. External facilitators enabled the feasibility of humanitarian work including employer or social flexibility, geographic proximity and shorter commitments, mitigating safety risks, and financial support. Multiple benefits realized from this work impacted participants personally and professionally through improved clinical skills and knowledge, sociocultural and language learning, and network building, among others. These positive incentives have been shown to be common among health volunteers, including improved physical and emotional wellbeing and that there may be a reciprocally beneficial relationship between these factors (26). Negative determinants shared similar themes, including internal sacrifices of family, career, personal stability and safety, and external barriers which limited effective clinical work including language differences and limited education, career development, and volunteer opportunities. Though not explicitly referenced by our sample, many of these negative determinants can have negative long-term consequences including burnout, career stagnation, and a psychological toll from working in high-stress environments. Both positive and negative determinants to migrant work have been well documented in other humanitarian contexts. A systematic review among health professionals working with migrants in Europe demonstrated the importance of sociocultural competence, adequate training and funding, and collaboration among interdisciplinary actors, but that health workers frequently reported exhaustion and reliance on coping mechanisms for emotionally stressful work (20). For those working in refugee settings similar to our sample, key challenges included linguistic and cultural barriers, lack of training, and feelings of helplessness among providers (27).

These findings contribute to understanding how to better address health worker shortages in migrant and humanitarian contexts. Our sample recommended dozens of tangible and feasible interventions to better recruit, support, and retain qualified health professionals caring for migrants. Such recommendations included early educational interventions for health professional students including integration of humanitarian curricula, formalizing partnerships between established academic institutions and humanitarian organizations, and better defining and rewarding career pathways through mentor, research, and clinical rotation opportunities. While the number of humanitarian-specific training opportunities has been increasing, there remains global inequality in access to these programs due to financial and geographical constraints (28). Furthermore, such concepts are rarely integrated into health professional education in a way that is mandatory or accessible to interested students (29). These approaches may be effective, as early exposure to medical care among vulnerable populations, service-learning, and partnerships between institutions and volunteer organizations have all been shown to increase health worker interest and participation in medical work among vulnerable populations (30, 31). Examples of such programs could include sponsored clinical rotations in humanitarian settings, mentored research projects with contextual experience through data collection, and courses on adaptation to medical practice in humanitarian care delivery, some of which have already been successfully implemented at health education training programs in the US, Democratic Republic of the Congo, Thailand, Canada, Australia, and New Zealand (32). Other strategies from our sample focused on raising awareness to recruit current health workers including advertising through professional societies, running medical outreach trips to raise awareness for migrant health disparities, information dissemination through publication and presentation to colleagues, and for organizations to expand research efforts and volunteer pools to include interdisciplinary fields. Potentially most impactful, our sample recommended formalizing collaborations between health employers and humanitarian organizations to mitigate one of the greatest barriers of career obligations and create bidirectional benefit for each institution. While such approaches have been proposed for humanitarianism, few have been actualized in practice (33). Finally, material and emotional support could increase participation including assisting with travel and other financial logistics, defining roles to mitigate uncertainty, and attentiveness to the mental health needs of health professionals (20).

There were several novel aspects to this study. First, we interviewed health professionals at the US-Mexico border, which despite the large increases in migrant populations and health needs, remains an understudied area compared with other refugee contexts. Since most humanitarian contexts overlap with under-resourced or LMIC health systems, our findings could have greater impact for resource mobilization from the US given proximity to deployable resources and US-based charities. Compared with the experiences of health professionals in other humanitarian settings, our sample revealed many unique determinants that were specific to working at the US-Mexico border. These included a moral obligation from US citizens to counteract restrictive immigration policies implemented by their own country, geographic proximity, and accessibility of short-term commitments. These differences suggest the importance of context-specific determinant assessments in recruiting and retaining humanitarian health professionals. Second, we intentionally recruited participants across two ports of entry at the US-Mexico border and selected for a diverse sample of providers across multiple organizations, health professional roles, humanitarian experiences including long and short-term commitments, and varied countries of origin and primary language. This diversity revealed differences across participant groups, including that Matamoros workers were longer-term, Reynosa participants more often benefited from employer-humanitarian organization partnership, physicians more often emphasized employer flexibility as a key facilitator, and those living close to the border were less likely to report sacrifices. There were unique challenges between short and long-term workers, such as career or family obligations and instability in social lives, respectively. Interestingly, criticisms of humanitarian aid and most benefits and motivators did not differ between participants of varied experience level, age, or health professional role, as might be expected. Despite the variation in our sample, common responses from participants demonstrated the universality of many determinants to humanitarian work and that recommended solutions could hold promise for other humanitarian contexts. Third, three of the health professionals in our study were part of the asylum seeker population they served. When such efforts have been made in other settings, unintentional negative consequences may arise, such as the case with refugee health workers in Uganda who faced increased discrimination, costly credentialing processes, and unclear clinical scope (34). These barriers were not identified by our sample, suggesting the influence of contextual factors, such as the heterogeneous nature of the migrant population themselves, as well as GRM’s hiring and credentialing process as an effective approach to equitable inclusion of asylum seeker health professionals. Finally, our sample enumerated 12 criticisms of humanitarian aid which limit organizations from optimizing care delivery. While challenges with humanitarian assistance have been hypothesized previously, limited in-depth evidence has evaluated the nuance behind and consequences of these critiques for health professionals working in the field (35, 36). Our qualitative evidence provides multifaceted and diverse accounts of how these challenges can have direct impacts on health professionals and the populations they serve.

Other findings from our study were notable. First, it was surprising that participants listed limited volunteer opportunities as a major barrier to humanitarian work. In light of evidence showing quantifiable health worker shortages in such contexts, there is a notable discrepancy in willingness to volunteer and opportunities to do so. Exploring the etiology for this discrepancy and piloting quality improvement efforts aimed at increasing the capacity of humanitarian organizations to accept health workers or improve recruitment materials could be valuable. Second, participants infrequently mentioned fear of indiscriminate violence or political pressure, which are common among humanitarian workers in other settings (27). This difference could be attributed to the relative stability of the US-Mexico border compared with other refugee contexts and freedom of movement and expression for US or Mexican nationals. Our sample did not mention rigidity of the health system as a barrier, despite this being a frequent challenge for health providers in other refugee settings (27). Given that GRM and MSF worked in small, mobile health teams, these operations may have increased flexibility for staff. Opposingly, a lack of rigidity may contribute to unstandardized protocols, treatment guidelines, or resource availability. Humanitarian organizations could benefit from determining the appropriate amount of operational structure to facilitate quality care for patients, while granting sufficient autonomy to health workers. Finally, our participants mentioned operational challenges including bureaucratic inefficiencies and a lack of standardized protocols. A persistent challenge for many health systems, such issues could be addressed through systematic approaches to organization, care delivery, and outcomes reporting through frameworks such as implementation science (37). More specifically, implementing evidence-based approaches could mitigate these challenges, including standardized clinical practice guidelines derived from organizational resources and forming humanitarian clusters based on the World Health Organization model through which organizations can collaborate (38).

### 4.1 Limitations

This study had several limitations. First, the qualitative nature of our methods limits generalizability to health workers outside of the US-Mexico border or even other ports of entry. Therefore, the challenges and determinants identified here may not fully represent those faced by health workers in all other global humanitarian settings. For example, the accessibility of volunteering, shorter-term commitments, and employer support as facilitators are likely unique to our sample and may not be available to those working in conflict zones or long-term development projects. We postulate that our findings may closely reflect those of other health workers working in border migrant settings but situated within more well-resourced environments. An additional limitation to generalizability was our smaller sample size with participants representing a limited number of humanitarian organizations. However, the in-depth explanations, varied roles, and extensive past humanitarian experiences from our sample suggest these results may be applicable in other settings, particularly when discussing determinants of border and migration humanitarianism. Second, our sample worked at the US-Mexico border for a limited amount of time among a select number of humanitarian organizations, which did not include all groups providing aid in Reynosa or Matamoros. This limitation could influence participant perspectives on operational challenges and intraorganizational dynamics. Our data collection periods were separated by approximately two and a half years, which could lead to different perspectives particularly in the US-Mexico border setting, where immigration policies are rapidly changing in nature and could have implications for migrant health. However, it was determined during analysis that major themes were shared between each location and that many of the providers who were interviewed in Reynosa had also worked in Matamoros over the preceding 3 years. Finally, our results only included health professionals. Though we included a diverse group of physicians, nurses, and logisticians among others, incorporating the perspectives of humanitarian organization administrators, government officials, or migrant patients themselves could produce more robust data.

## 5 Conclusion

Health professionals working with asylum seekers and refugees at the US-Mexico border were able to identify common positive and negative determinants to delivering health services to migrants in humanitarian settings, as well as recommendations to address health worker shortages. Determinants included incentivizing motivations, facilitators, and benefits, alongside disincentivizing sacrifices, barriers, and challenges. Recommendations reflected feasible and early intervention strategies for recruiting health professionals including integration of clinical and research opportunities into health professional education, collaboration between health institutions and humanitarian organizations to reduce employment-related barriers, and increasing transparency and professional support services to clarify the process for working with humanitarian organizations.

## Data Availability

The raw data supporting the conclusions of this article will be made available by the authors, without undue reservation.
