# Supplementary material for: Determinants for the humanitarian workforce in migrant health at the US-Mexico border: optimizing learning from health professionals in Matamoros and Reynosa, Mexico
Source: Front Public Health. 2024 Oct 10;12:1447054. doi: 10.3389/fpubh.2024.1447054 (PMC11499189; doi:10.3389/fpubh.2024.1447054)
Supplement: Supplementary file 1 [file Table_1.DOCX]

Supplementary material 1: Semi-structured interview script among health professionals in Matamoros and Reynosa, Mexico

Have you read the informed consent? (yes/no)

Do you have any questions at this time? (yes/no)

Would you like to participate? (yes/no)

**Demographic questions**

1. How old are you?
2. What is your gender?
3. Where were you born? Where do you currently live?
4. What is your highest level of education?
5. What is your role? (doctor, nurse, social worker, physician assistant, other)
6. Could you describe a typical day for you?
7. Have you provided health care services to asylum seekers?
8. How long have you provided the service?
9. Where did you provide your services?
10. Do you work with an interdisciplinary team? (health or other disciplines)
11. If interdisciplinary, what other professionals or staff were involved?
12. Have you conducted brigades or participated in humanitarian or international medicine programs before working with asylum seekers? (including with vulnerable populations)?

**Evaluate determinants to working with asylum seekers and its effect on healthcare providers’ perspectives and capacity**

1. What reasons did you have for deciding to work with this population?
2. How or why did you decide to work with this population of asylum seekers?
3. Could you describe how you got there? (On a brigade or worked/lived there for a long time)
4. If on a mission trip, where do the funds and logistical support for the brigade come from?
5. What were the biggest personal “sacrifices” you had to make to work here?
6. Has your work with this community changed your opinion of this population?
7. If yes, how has it changed?
8. Would you recommend a colleague to work in this area or with a population of asylum seekers?
9. What do you think could be done to motivate other doctors to work with asylum seekers?
10. After this experience, are you more or less willing to work with vulnerable or marginalized populations in this future?
11. Has your perspective on the best way to provide health services to vulnerable populations changed? How?
12. Do you feel that asylum seekers appreciate your work and assistance?
13. In the care or consultation you provide them, how easy or difficult is it to obtain health information from this community? (Is it easy to talk about health issues with these patients?)
14. Did you receive any type of training specifically for providing health care to refugees or asylum seekers?
15. Could you describe the training or preparation you underwent?
16. Are specific strategies required to serve asylum seekers, different from those required for other patients? What strategies?
17. Can you describe the coordination process among the various organizations involved in the care of asylum seekers? How do they coordinate?

**Provide tangible recommendations on how to increase the participation of healthcare providers in humanitarian medicine.**

1. What can be done to help improve the participation of other healthcare providers in working with migrants and asylum seekers?
2. Based on your experience, what are some of the main barriers to humanitarian medicine in general?
