# Supplementary material for: Determinants for the humanitarian workforce in migrant health at the US-Mexico border: optimizing learning from health professionals in Matamoros and Reynosa, Mexico
Source: Front Public Health. 2024 Oct 10;12:1447054. doi: 10.3389/fpubh.2024.1447054 (PMC11499189; doi:10.3389/fpubh.2024.1447054)
Supplement: Supplementary file 2 [file Table_2.DOCX]

Supplementary material 2: Validated codebook used to code transcripts of interviews among health professionals in Matamoros and Reynosa, Mexico

Codes

| Name | Description |
| --- | --- |
| Benefits | Gains for volunteer work |
| Career Advancement | Promotion or professional development opportunities that come from this work |
| Cultural Learning | Learning about other cultures or people from diverse backgrounds, including family dynamics, what patients expect from health care, and about countries of origin or migration |
| Emotional Connection | Gaining a deeper sense of humility or understanding of other people's conditions. Also, if participant mentions enjoyment, self-worth, or joy from working with this patient population in humanitarian contexts |
| Empowerment | Participants benefit from seeing other volunteers be changed from their first time experiencing a humanitarian and service mission |
| Network | Increased relationship building with professional contacts, new friends from humanitarian work experiences |
| New opinions |  |
| New Skills | Learning new approaches (either at systems level or individual clinical skills) from being involved in this work) |
| Travel | Ability to travel the world or sense of adventure that comes from this work |
| Challenges and Criticisms of humanitarian aid |  |
| Bad Metrics | A lack of quality metrics on which to gauge an organization and services being rendered |
| Balancing community needs w realism |  |
| Bureaucracy | Red tape, bureaucracy, and other measures that prevent quality work from being done in humanitarian efforts |
| Burnout |  |
| Funding | Sustainability difficulties which depend on funding streams of the organization or institution |
| Lower Quality | Mentioning that many resources and money go into efforts that ultimately do little good for the populations they are intended to serve. "So much goes into putting the wrong people in the wrong place." OR "that lower quality of care is being delivered through humanitarian aid systems |
| Minimize capacity_turf wars or bad NGO coordination | Tendency of humanitarian aid groups to strip local capacity or health systems and ability to help themselves |
| Protocols | Challenge of determining the standard of care or local treatment patterns when working in a new environment |
| Show Appearance | The focus of some humanitarian efforts to be more focused on showing off rather than doing good work |
| Usefulness | Individual's Lack of skills when becoming involved in humanitarian aid work, or intentional waiting until they have skills to volunteer |
| Demographics | Demographic variables that appear at the beginning of each interview |
| Age | Age of the participant at the time of the interview |
| Country of Origin | For all English speakers this will be the United States. For asylum seekers or health providers this will vary. |
| Current Occupation | Career outside of volunteering |
| Education | Highest Degree of education attained |
| Gender | Gender of the interview participant |
| GRM work | Description of the work volunteer does with GRM |
| Previous Asylum Experience | If the volunteer has worked with asylum seekers, refugees, or other migrant populations in the past, either in Reynosa, Matamoros, or another context. This code should automatically trigger a double code with previous humanitarian experience. |
| Previous Humanitarian Experience | Does the participant have previous public health experience that caused them to continue volunteering? |
| Motivators and Facilitators | Positive drivers, motivations that the interviewee reports for participating in humanitarian aid work |
| Desire to care for Vulnerable populations | An intrinsic motivation to care not just for others, but specifically vulnerable populations |
| Family Upbringing | Did their upbringing include values or role models that inspired them to do this work? |
| Frustrations with the US health system | Desire to remove the frustrations of operating in a US health system including insurance, electronic health record, and prescribing limitations. This code will mainly be used when discussing frustrations at one's home institution and how domestic (US) patients are treated. Different from USA obligation, which hits on more of a theoretical need to help because of government/political policy. |
| Future Participation | Does the participant have previous public health experience that caused them to continue volunteering? Or did this experience cause them to be more involved in the future? |
| Humanitarian distress | Witnessing the terrible conditions, human suffering, and human rights violations which people are subjected to. Examples could include terrible living conditions, disaster effects, 9/11, witnessing of human suffering |
| Justice | If the participant states a belief in some greater call towards justice, obligation to help, or caring for others |
| Personal Hardships | If participant mentions their own hardships as being motivation to help others through similarly difficult times |
| Recommendations from colleagues | Became involved in humanitarian work from encouragement or opportunities made from other professional or social relationships |
| Religion | If participant mentions faith or religion as a primary motivator for involvement in this work |
| Sociocultural Learning_language acquisition | The desire to learn more about other people and their culture |
| Work required or facilitated | Person occupies a job/role that requires them to work in these contexts |
| Other | Text that otherwise does not fit into the above categories |
| Recommended Solutions | Ideas from the participant to create more opportunities and interest in humanitarian aid |
| Collaboration and Recruitment | References to the idea of bringing as many people into this work as possible independent of their training or background. this can include reaching out to the "non-typical" actors in humanitarian contexts (anyone outside of trauma, emergency medicine) including OB/GYN. nonmedical like social workers, interdisciplinary staff, and psychosocial support |
| Creativity | Some mentioned creative options for making humanitarians work possible including changing their work schedule to accommodate, negotiating contract to protect that time, or becoming involved in research/education projects around these issues |
| Demonstration | Showing opportunities to be involved and the need that exists through education and exposure. Includes more opportunities for students to learn about and become involved in this work. Demonstrating opportunities for involvement |
| Discernment | Health providers should genuinely reflect on if they would enjoy this work despite the difficulties and stress. Because not doing so could result in frustrations that result in burnout and pessimism |
| In-country partners | Mention of increased effectiveness for NGOs working with people and infrastructure already within a country |
| Institutionally available | Allowing for medical professions to provide time off for opportunities from their work institution, potentially even compensated, or even having these chances available through their home health system |
| Logistical Support | Making the process more accessible and affordable by providing housing, free flights, transportation, medica insurance, and other logistics from either the volunteering organization or home institution |
| Mental Health support | Making sure there is appropriate mental health and psychosocial support networks for humanitarian aid workers |
| Skills building and career pathways | what individuals did to intentionally build skills before, or while being involved in humanitarian work. can include medical certifications, taking time off while inexperienced. research, formal program. |
| Sacrifices and barriers | Participant mentions personal sacrifices or barriers which make it harder to become involved in or sustain this work |
| Career Obligations | If doing this work negatively impacts career opportunities including promotion decisions or professional development. Also, should include having to take significant time off work or delaying participation due to work obligations |
| Emotional Difficulties | If doing this work has an emotional toll from witnessing pain and suffering |
| Family | Any mention of difficulty related to family, including time away from them, struggles with partners, or family members who do not understand their jobs |
| Language | Difficulty in speaking different languages between other aid workers and/or patients |
| Limited Autonomy | If participant has less control over their own schedule or time given their involvement in this work |
| Limited knowledge and Education | The health professional simply does not know that these opportunities exist. Or that universities and graduate schools do not show their students what humanitarian aid programs exist, leading to limited knowledge and education on these topics |
| Limited Opportunities | Not knowing how or when to become involved in humanitarian work. Limited avenues for volunteering or working for an organization |
| No sacrifice |  |
| Safety and comfort | Stresses or difficulties that come from safety concerns including risk of death or injury from this work. can also include leaving the comfort of one's home or place of work to become involved in stressful volunteering situations. |
| Salary | Lower salary, pay cuts, or having to be a volunteer which come from humanitarian work |
| US Border Involvement Specifics | What are the specific characteristics of Reynosa and GRM that allow to encourage participants to do this work? |
| Accessibility of helping | Comments that it is easier to help at the US border based on the scope. compared with other refugee or disaster response setting around the world |
| Lack of Others | The need to help because there is a lack of international response of coordination from UHNCR, WHO, United Nations, governments, or other common large-scale players who would typically help in a humanitarian |
| Longevity | Since the US border crisis is a chronic problem, it allows more longitudinal partnership between health institutions and on the ground partners. |
| short-term commitment | Volunteers can come for a few weeks, which does not require full commitment |
| USA obligation | Ethical obligations for US citizen to be involved in the border solutions since their government is contributing to the problem. This should also involve frustrations with how US treats international persons including migrant. |
| Value Pairing | The work at the border ties nicely with other work being done by the academic center |
