# Supplementary material for: Determinants for the humanitarian workforce in migrant health at the US-Mexico border: optimizing learning from health professionals in Matamoros and Reynosa, Mexico
Source: Front Public Health. 2024 Oct 10;12:1447054. doi: 10.3389/fpubh.2024.1447054 (PMC11499189; doi:10.3389/fpubh.2024.1447054)
Supplement: Supplementary file 3 [file Table_3.DOCX]

Supplemental material 3: Extended table of recommended solutions to handle challenges of humanitarian assistance among migrants in Reynosa and Matamoros, Mexico

| Sacrifices, barriers, or challenges addressed | Potential solutions | Representative quote |
| --- | --- | --- |
| Bureaucracy and prioritization of appearance over outcome | Emphasize focus on individual clinical encounters | “There so much bureaucracy and red tape that by the time these international groups arrive, most of the response has been done already locally. So I think the field needs to focus more on building local response mechanisms in places that are highly prone to disasters, not about sending academic medical teams from the US when it is already too late to do anything. Otherwise it becomes a show and photo-op. The most effective organizations are ones with community engagement, who have already established partners. They have mechanisms to be safe, supply chains established, and all the things it would take a foreign team forever to set up” -Nurse |
|  | Incorporate organizational management skills when training volunteers |  |
|  | Foster longitudinal collaborations with in-country partners |  |
|  | Build local capacity |  |
|  | Screen out volunteers with motivations not aligned with organizational values |  |
|  | Separate organizational promotion activities from clinical humanitarian responsibilities |  |
| Burnout and secondary trauma | Provide mental health support services to volunteers | “There definitely needs to be a piece of this work where you’re in a community where people acknowledge the secondary trauma that can happen. I actually trained to be a chaplain for the disaster team. And one of the experiences we had on assignment was so awful, I felt that support was needed and appreciated.” -Physician |
|  | Reduce nervousness through clear communication channels |  |
|  | Allow decompression and reflection opportunities following traumatic experiences |  |
|  | Consider spiritual support services including chaplains |  |
|  | Foster strong communities where secondary trauma can be processed |  |
| Career obligations | Institutionalize volunteer opportunities through employers | “I don’t create the motivation, rather facilitate it from a professional perspective. If I can wedge out a bit of our employees’ time and professional responsibilities and keep them getting paid, I can create a safety net to bring in all kinds of people who normally wouldn’t work in these settings.” -Nurse |
|  | Promote leadership which will dedicate time to humanitarian work |  |
|  | Select a job in humanitarianism |  |
| Emotional burden from witnessing suffering | Undergo discernment for emotional resilience prior to volunteering | “Not everyone should be here helping. It is a difficult decision to become involved and not just anyone should be trying to manage these types of situations. It’s a strong emotional burden, though I’ve been able to withstand it okay.” -Social worker |
|  | Do not require participation from those unready or able |  |
|  | Intentionally recruit those with appropriate skills and disposition |  |
| Family and social obligations | Pursue humanitarian work when commitments are minimal (for most, older or younger in career) | “I’ve always wanted to explore a humanitarian project, but timing wise, I couldn’t do this until my kids were out of the house. And then this opportunity appeared, where there was a need in my own backyard, so I jumped at it.” -Physician |
|  | Seek similarly-minded social groups for support |  |
|  | Select opportunities which mitigate tension between family and work |  |
|  | Involve families in work, where appropriate |  |
| Financial burden | Provide salary coverage | “The big barrier for me was financial assistance to come here. So that’s a huge incentive if things like airfare or housing can be provided. It could be done in conjunction with some sort of commitment, like if we pay for X amount of your trip, then you’ll give us three week of volunteer time per year.” -Nurse |
|  | Offer logistical support in covering travel or lodging expenses |  |
|  | Formalize volunteer time commitments for logistical support |  |
| Forgoing personal vacation | Integrate humanitarianism into paid-time-off | “I was fortunate in the past to get a two-week time off to get to the Bahamas for disaster work, because my hospital is so committed to dong global health and some of us are able to go with the blessing of the department. Otherwise you’d have to take vacation time.” -Nurse |
|  | Provide enjoyable activities in the local community |  |
|  | Emphasize travel benefits |  |
| Funding limitations | Facilitate research to increase grant opportunities | “Gathering data should be happening, because no one’s going to know about the needs. You’re not going to get the funding you need. It’s like there’s no information on what’s going on.” -Nurse |
|  | Intentionally seek funding with minimal limitations or specific project plans |  |
|  | Consider partnership with industry or charitable fundraising organizations |  |
| Ignorance to health need or humanitarian crises | Write or publish stories, blogs, or research about humanitarian need | “I think it would be great to do medical campaigns, show people what is really happening here. Because most doctors don’t actually know what is occurring. They just say ‘oh, they are migrants, but they already have help,’ when really that’s not true.” -Physician |
|  | Demonstrate need to friends and colleagues with stories and photos |  |
|  | Offer short-term medical outreach campaigns |  |
| Instability from a nomadic lifestyle of constant travel | Seek local humanitarian opportunities including US-Mexico border | “By taking a job with this organization, they are the ones giving me permission to go on each assignment. It helps to work with just one organization and have control over my travel.” -Nurse |
|  | Consider longer-term volunteering or full-time humanitarian employment |  |
| Interorganizational conflict | Define clear roles based on capacity | “The organizations have a clear distinction in what their doing. One handles physical care while the other does mental health. They sometimes have patients that overlap and seem to do a good job of collaborating when needed and sharing services.” -Nurse |
|  | Foster working relationship with other regional organizations |  |
|  | Schedule regular meetings to share updates and challenges |  |
| Language barriers | Learn language skills prior to volunteering | “One improvement would be if a translator was with us everywhere who know the everyday clinic operations. Our volunteers have different speaking capabilities. Some know Spanish and less speak Haitian Creole, so we often rely on the migrants to translate for a bunch of strangers on both ends.” -EMT |
|  | Limit volunteerism to areas of language competence |  |
|  | Increase interpreter services |  |
| Limited career rewards | Provide incentives for humanitarian work | “I don’t think there’s monetary rewards in humanitarian work. Being prestigious, well-known, or famous for doing good work, getting some sort of award or writing in a journal or book about your experience, I feel like that could incentivize people.” -EMT |
|  | Offer research, grant, and promotion opportunities in humanitarianism |  |
|  | Highlight pathways for career recognition, awards |  |
| Limited knowledge, education, and career development pathways | Integrate humanitarianism into medical education | “It needs to be readily available in education. Having a professor that has done humanitarian work, who students can speak to and hear from someone whose actually done it and has knowledge on the subject. And then if students want to, opportunities could be created. If an organization partnered with a school, they could schedule it, like you’re going to be there for the next two weeks. It would give that student a very real, hands-on experience to get their feet wet. If there was more of an emphasis on it in schooling, it wouldn't be such an unknown path forward or difficult thing to do.” -EMT |
|  | Hiring health education faulty with humanitarian expertise |  |
|  | Collaboration between health education schools and humanitarian organizations |  |
|  | Design curricula and service-learning opportunities for health students |  |
|  | Website transparency |  |
| Limited opportunities | Create organizational partnerships | “One way is to increase recruitment. Something like providing lectures, or contacting organizations like the American college or any national society and letting people know these opportunities are available. Because I know many health professionals who want to do this work but they don’t know about how to go about it or the right organizations to contact. Even newspapers, or paying to get a marketer. And for us, doing all we can to get the word out to others we work with. -Nurse |
|  | Promote volunteering with smaller organizations |  |
|  | Recruit through national societies |  |
|  | Present humanitarian experiences to social networks |  |
|  | Actively recruit colleagues with well-suited skills |  |
| Minimization of local capacity | Intentionality towards capacity building efforts | “One thing is if someone from the humanitarian organization could come to our hospital and give a talk about their work? I think building relationships between these groups and the major hospitals to allow for that kind of exchange is beneficial. Research efforts too, where if you present you have someone from the community also discuss their experience.” -Nurse |
|  | Bidirectional exchange and learning opportunities |  |
|  | Equitable co-design |  |
| Poor data collection metrics | Partner with academic centers to improve research capacity | “There’s no data for what exactly is happening here. That’s why doing some of these interviews is such a game changer, but there needs to be more research. You could quantify how much money is being spent on treatments to take care of people who don’t have access to clean water in this population. Or interview those who have been here awhile to figure out what is the situation, what are the barriers and how can we fix them?” -Nurse |
|  | Research impact of implementing public health measures |  |
|  | Conduct mixed-methods health services or implementation research |  |
|  | Leverage clinical records to quantity disease burdens |  |
| Protocols being different or unstandardized | Prepare volunteers for new adaptations to treatment algorithms | “I feel like clinical guidelines with a clear pathway to look up anything you are uncertain about would be helpful, because we see so many consistent things. If you’re lost, you can look up the condition and it gives treatments with doses, which would be helpful since we don’t know all the resources here.” -Nurse |
|  | Distribute context-appropriate clinical guidelines to staff |  |
|  | Standardize protocols according to context |  |
| Provider feelings of uselessness | Take time to develop proper clinical and humanitarian skills before volunteering | “When I started my new job, I felt that I finally developed a baseline skill set in clinical care. But that’s one thing, and the entire other skill set in the humanitarian sphere is a whole other. I started to volunteer with our disaster response team.” -Nurse |
|  | Align volunteer skills with community needs |  |
|  | Provide resources for adequate care delivery |  |
| Risks to safety and comfort | Well designed safety protocols | “One improvement would be more transparency in the process of operations, what a typical day looks like with responsibilities. I think the unknown scares people away from it. If you say I’m going out of the country for a humanitarian medical trip, people think it’s crazy. They think its so unsafe. If we can make the work more transparent, especially through the websites of these organizations, it would definitely get more interest.” -Nurse |
|  | Transparency on day-to-day activities as a volunteer |  |
|  | Explanation of role expectations prior to involvement |  |
|  | Working in less risky environments |  |
| Tension between community expectations and organization capacity | Well-designed needs assessments prior to partnership | “When I’m working under the nursing fellowship program, I’m always looking to match my partners’ needs first. I’m asking ‘what do they need?’ And when they share that need, for example they’ll say ‘a research instructor for the Master’s students,’ I’ll go back to my group to see if we can provide that resource.” -Nurse |
|  | Involve community in program planning |  |
|  | Make institutional knowledge and feasible resources transparent where appropriate |  |
| Time away from work as burdensome on clinical partners | Select a practice with multiple and flexible partners who can cover | “You have to be working with a group that can cover you when you’re gone. If global health is really important to someone, they’ll look for jobs where it’s not a burden. For example you’re on call the month of September so you can’t go, but October becomes your month to do global health.” -Physician |
|  | Pursue shorter-term volunteer commitments |  |
|  | Negotiate time for humanitarian work into one’s contract |  |

Table 4 footnote: Barriers in Table 4 were intentionally selected to reflect personal barriers for health professionals and those relating to the most commonly mentioned recruitment strategies. A full list of sacrifices, barriers, and challenges with corresponding solutions and representative quotes is available in Supplemental material 3.
