# Supplementary material for: Determinants for the humanitarian workforce in migrant health at the US-Mexico border: optimizing learning from health professionals in Matamoros and Reynosa, Mexico
Source: Front Public Health. 2024 Oct 10;12:1447054. doi: 10.3389/fpubh.2024.1447054 (PMC11499189; doi:10.3389/fpubh.2024.1447054)
Supplement: Supplementary file 4 [file Table_4.DOCX]

June 10, 2024

Bruce Struminger, MD, MA

Topic Editor, *Frontiers in Public Health*

University of New Mexico School of Medicine

2500 Marble Ave NE

Albuquerque, NM 87106

Dear Editors:

We are pleased to submit our manuscript entitled, “Determinants for the humanitarian workforce in refugee health at the US-Mexico border: optimizing learning from health professionals in Matamoros and Reynosa, Mexico” as original research to the Learning Interventions and Training: Providing Support during Health Emergencies special issue of *Frontiers in Public Health*.

Health workforce shortages remain one of the most pressing problems to optimize health systems in humanitarian settings, particularly among migrant and refugee populations. There is limited research on factors which enhance or inhibit a health professional’s likelihood of participating in humanitarian work, particularly at the US-Mexico border. There, millions of migrants annually seek entry into the United States and receive health services from humanitarian organizations while residing in non-permanent encampments. Our qualitative study evaluates determinants for health professionals working in humanitarian migrant contexts, as well as recommendations to address health workforce shortages. Specifically, we present common motivations, facilitators, barriers, sacrifices, challenges, and solutions to increase health professional involvement in migrant health delivery at two major ports of entry at the US-Mexico border: Matamoros and Reynosa, Mexico. While many studies have evaluated health professional perspectives regarding global and rural health, ours introduces novel findings specific to the humanitarian needs of the US-Mexico border, incorporates a diverse sample of participants comprising seven health professions and five countries, and evaluates universal challenges of humanitarian aid from the perspective of experienced clinicians.

Our findings are novel and impactful, and we believe they are of interest to medical, health education and policy, public health, and health services research community. Our hope is that these findings can help to empower humanitarian organizations, medical educators, and health employers to facilitate quality involvement in humanitarian migrant work. This manuscript has not been previously published and is not under consideration in any other journal. All authors contributed to the manuscript, including reviewing and editing the final version. Thank you for the opportunity to submit our work to *Frontiers in Public Health,* and we look forward to hearing from you.

Sincerely,


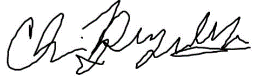


Christopher W. Reynolds

Department of Surgery, University of Michigan

1500 E. Medical Center Dr.

Ann Arbor, MI, 48109

[chwre@med.umich.edu](mailto:chwre@med.umich.edu)

1-302-377-9925
